# What do we mean with sound semantics, exactly? A survey of taxonomies and ontologies of everyday sounds

**DOI:** 10.3389/fpsyg.2022.964209

**Published:** 2022-09-29

**Authors:** Bruno L. Giordano, Ricardo de Miranda Azevedo, Yenisel Plasencia-Calaña, Elia Formisano, Michel Dumontier

**Affiliations:** ^1^Institut des Neurosciences de La Timone, CNRS UMR 7289 – Université Aix-Marseille, Marseille, France; ^2^Faculty of Science and Engineering, Institute of Data Science, Maastricht University, Maastricht, Netherlands; ^3^Faculty of Science and Engineering, BISS (Brightlands Institute for Smart Society) Institute, Maastricht University, Maastricht, Netherlands; ^4^Department of Cognitive Neuroscience, Faculty of Psychology and Neuroscience, Maastricht University, Maastricht, Netherlands

**Keywords:** natural sounds, ontologies, knowledge-representation systems, semantics, sound source, sensation, acoustic signal, onomatopoeia

## Abstract

Taxonomies and ontologies for the characterization of everyday sounds have been developed in several research fields, including auditory cognition, soundscape research, artificial hearing, sound design, and medicine. Here, we surveyed 36 of such knowledge organization systems, which we identified through a systematic literature search. To evaluate the semantic domains covered by these systems within a homogeneous framework, we introduced a comprehensive set of verbal sound descriptors (sound source properties; attributes of sensation; sound signal descriptors; onomatopoeias; music genres), which we used to manually label the surveyed descriptor classes. We reveal that most taxonomies and ontologies were developed to characterize higher-level semantic relations between sound sources in terms of the sound-generating objects and actions involved (what/how), or in terms of the environmental context (where). This indicates the current lack of a comprehensive ontology of everyday sounds that covers simultaneously all semantic aspects of the relation between sounds. Such an ontology may have a wide range of applications and purposes, ranging from extending our scientific knowledge of auditory processes in the real world, to developing artificial hearing systems.

## Introduction

Sounds, i.e. the acoustic signals that populate our environment, are ubiquitous in everyday life. Humans and animals produce sounds to communicate (e.g. speech, vocalizations) and as a consequence of their actions and of their interaction with the environment (e.g. walking, flapping wings). In addition, many manufactured objects and devices that we use daily produce sounds through a variety of mechanisms and processes (e.g. hammering, combustion engines). The development of knowledge organization schemes for the characterization and systematization of natural sounds and of their relations has been an object of interest in several research fields, including cognitive and behavioural psychology, machine sound classification, sound design, music theory, soundscape analysis and medicine. Here, we survey this multifaceted literature to assess the state-of-the-art and to aid the future development of comprehensive everyday sound ontologies.

The frameworks for the description of everyday sounds available in the literature differ greatly in terms of their methodology and complexity (Guastavino, 2018). In the simplest cases, sound classifications are obtained by grouping sounds according to some settled attribute (e.g., voice vs. tool sounds, Lewis et al., 2005; urban vs. rural soundscape contexts, De Coensel et al., 2003; a classification is a set of groups that distinguishes different kinds of things). In other more complex cases, taxonomies are provided in which sounds are arranged in nested, cumulative hierarchies, extending to certain depths (Gaver, 1993; a taxonomy typically follows a hierarchical organization based on a shared relation, e.g., is-a/subclass of). Finally, in a few cases, ontologies are proposed that describe attributes and properties of sounds as well as the connection between them in an any-to-any direction and in a machine-readable and machine-interpretable format (Cherny et al., 2016; an ontology focuses on the description of entities and their relations, and on making a computational representation of this knowledge accessible to automated reasoning). Here, we focused on taxonomies and ontologies with the goal of systematically surveying the state of the art in the characterization of sound semantics from both a quantitative and qualitative perspective.

Taxonomies and ontologies served different purposes in different research fields. In *auditory cognition*, they have aided research on the factors driving human perception of complex sounds (e.g., Gygi et al., 2007; Lindborg, 2016). Here, theoretically-driven sound taxonomies have been proposed based on psychoacoustic dimensions (pitch, loudness, timbre) or on the physical properties of sound-generating objects (e.g., solid, liquid, gas phases) and related physical processes (e.g., impacts, explosions, flows; Gaver, 1993, p. 1). Alternatively, sound taxonomies have been derived within a data-driven framework from the results of sound perception experiments involving various behavioral tasks (Gygi et al., 2007). Within the domain of *soundscape* research (Southworth, 1969; Schafer, 1977; Guastavino, 2018), taxonomies have been developed to describe and characterize complex acoustic scenes in different contexts (e.g., urban, nature) and locations (e.g., restaurant, household), their perception (Lindborg, 2016), and their impact on quality-of-life metrics such as noise pollution. In the field of *sound design*, description schemes are used to structure the metadata of large sound databases and for automated audio (i.e., recorded, transmitted or reproduced sound) retrieval, for example, to select sound effects for the digital content industry (e.g., film and video game industries) (Cherny et al., 2016). *Machine learning and artificial hearing* are other relevant research fields in which sound description schemes have been developed. This field is rapidly expanding, also thanks to recent breakthroughs and techniques such as deep neural networks (DNN), which achieved near-human performance for different sound recognition tasks (Hershey et al., 2017). In this research field, sound description schemes are developed to organize labels for training DNNs and other supervised algorithms (Gemmeke et al., 2017) and to assess the algorithms' performance. Finally, taxonomies have been developed to describe in great detail specific classes of sound sources, e.g., in *music theory* (i.e., the discipline encompassing the methods and concepts used to compose music, where music is defined as the “vocal or instrumental sounds—or both—combined in such a way as to produce beauty of form, harmony, and expression of emotion”—Oxford Languages dictionary) to systematically classify musical instruments based on the mechanisms of sound production (von Hornbostel and Sachs, 1961) or in *medicine* to distinguish physiological from pathological sounds and assist in the diagnostic procedure (Prystupa, 2013).

The remainder of the article describes the work we conducted: (i) to identify publications reporting on taxonomies or ontologies of sounds; (ii) to map each manuscript/scheme to a specific research field; (iii) to quantify metrics of these knowledge organization systems (e.g., total number of classes/levels) and (iv) to label each class in the systems according to their sound descriptors type (e.g., acoustics; sound quality, sound-source semantics, or other). We then briefly discuss representative publications in each research field, with the goal of highlighting their distinctive aspects. Finally, we discuss our findings critically and suggest general guidelines for the future development of comprehensive sound ontologies. The data analyzed in this survey, as well as an implementation of each of the identified taxonomies and ontologies using the Web Ontology Language (OWL), are made available in a public repository (see Data and code availability, below).

## Methods

### Search and selection strategy

From October 2020 to January 2021, we used two approaches to retrieve existing taxonomies and ontologies of sounds:

(a) Two systematic searches on the databases *Google Scholar* and *Web of Science* were performed on December 8th 2020. The following terms were included in the search: (i)“*Audio*”; “*Sounds*”; (ii) “*Taxonomy*”'; “*Ontology*.” (i) and (ii) were separated by the Boolean term “*AND,”* and the terms within (i) and (ii) were separated by the Boolean term “*OR*.”
(b) References in key publications about sound taxonomies and ontologies.

Records were eligible for the survey if the full specification of the taxonomy/ontology was available either as a computer file (TTL, JSON, OWL), or in the manuscript. Records were excluded if the sound description scheme was a simple classification (i.e., a set of terms), or if the scheme did not provide all class headings (e.g., a dendrogram with subordinate class headings only). Taxonomies specific to music (e.g., music genres, Pachet and Cazaly, 2017; musical instruments, von Hornbostel and Sachs, 1961) were not considered to keep the review focused on natural sounds and avoid results heavily biased toward the music domain. Note that this criterion did not result in the complete elimination of music-related descriptors from our analyses because part of the selected taxonomies did include music genre and musical instrument classes (e.g., Gemmeke et al., 2017). All retrieved records were combined for duplicates removal, using R statistics package dplyr (Hadley et al., 2022), and further by manually removing additional duplicates not detected using dplyr. Records were selected by authors RdMA and BLG, first based on a screening of the abstracts, and second based on the full text. The list and characteristics of the records selected for this review are reported in Table 1.

**Table 1:** Sound categorization schemes selected for this review, and their characteristics.

**References**	**Title (ID)**	**Research field**	**Type**	**Reasoning**	**N** **classes**
Gaver (1993)	What in the world we hear? An ecological approach to auditory event perception	Auditory cognition	Taxonomy	Theoretical	56
Guastavino (2007)	Categorization of Environmental Sounds	Auditory cognition	Taxonomy	Data-driven	17
Gygi et al. (2007)	Similarity and categorization of environmental sounds	Auditory cognition	Taxonomy	Data-driven	67
Houix et al. (2012)	A lexical analysis of environmental sound categories.	Auditory cognition	Taxonomy	Data-driven	12
Lemaitre and Heller (2013)	Evidence for a basic level in a taxonomy of everyday action sounds	Auditory cognition	Taxonomy	Data-driven	66
Guyot et al. (2017)	Identification of categories of liquid sounds	Auditory cognition	Taxonomy	Data-driven	22
Bones et al. (2018)	Sound Categories: Category Formation and Evidence-Based Taxonomies (Dogs)	Auditory cognition	Taxonomy	Data-driven	6
Bones et al. (2018)	Sound Categories: Category Formation and Evidence-Based Taxonomies (Engines)	Auditory cognition	Taxonomy	Data-driven	13
Bones et al. (2018)	Sound Categories: Category Formation and Evidence-Based Taxonomies (Man-made)	Auditory cognition	Taxonomy	Data-driven	28
Bones et al. (2018)	Sound Categories: Category Formation and Evidence-Based Taxonomies (Nature)	Auditory cognition	Taxonomy	Data-driven	21
Bones et al. (2018)	Sound Categories: Category Formation and Evidence-Based Taxonomies (Soundscape)	Auditory cognition	Taxonomy	Data-driven	50
Schafer (1977)	Our sonic environment and the soundscape, the tuning of the world	Soundscape	Taxonomy	Theoretical	215
Brown et al. (2011)	Toward standardization in soundscape preference assessment	Soundscape	Taxonomy	Theoretical	47
Salamon et al. (2014)	A Dataset and Taxonomy for Urban Sound Research	Soundscape	Taxonomy	Data-driven	166
Huang and Kang (2015)	The sound environment and soundscape preservation in historic city centers—the case study of Lhasa	Soundscape	Taxonomy	Theoretical	21
Lindborg (2016)	A taxonomy of sound sources in restaurants	Soundscape	Taxonomy	Data-driven	58
Trudeau and Guastavino (2018)	Classifying soundscapes using a multifaceted taxonomy	Soundscape	Taxonomy	Theoretical	7
Lafay et al. (2019)	Investigating the perception of soundscapes through acoustic scene simulation (Mechanical events)	Soundscape	Taxonomy	Theoretical	35
Lafay et al. (2019)	Investigating the perception of soundscapes through acoustic scene simulation (Non-mechanical events)	Soundscape	Taxonomy	Theoretical	37
Lafay et al. (2019)	Investigating the perception of soundscapes through acoustic scene simulation (Textures)	Soundscape	Taxonomy	Theoretical	32
Nakatani and Okuno (1998)	Sound Ontology for Computational Auditory Scene Analysis.	Machine hearing	Ontology	Theoretical	43
Casey (2001)	General sound classification and similarity in MPEG-7	Machine hearing	Taxonomy	Theoretical	29
Gerhard (2000)	Audio Signal Classification	Machine hearing	Taxonomy	Theoretical	47
Mitrovic et al. (2010)	Features for content-based audio retrieval.	Machine hearing	Taxonomy	Theoretical	87
Perperis et al. (2011)	Multimodal and ontology-based fusion approaches of audio and visual processing for violence detection in movies	Machine hearing	Ontology	Theoretical	13
Gemmeke et al. (2017)	Audio Set: An ontology and human-labeled dataset for audio events	Machine hearing	Taxonomy	Data-driven	632
Jiménez et al. (2018)	Sound event classification using ontology-based neural networks (MSoS)	Machine hearing	Taxonomy	Data-driven	18
Jiménez et al. (2018)	Sound event classification using ontology-based neural networks (US8K)	Machine hearing	Taxonomy	Data-driven	15
Miranda et al. (2000)	Categorizing complex dynamic sounds	Sound design	Taxonomy	Data-driven	28
Cherny et al. (2016)	An approach for structuring sound sample libraries using ontology	Sound design	Ontology	Data-driven	288
Baldan et al. (2017)	The sound design toolkit	Sound design	Taxonomy	Data-driven	37
Moffat et al. (2017)	Unsupervised Taxonomy of Sound Effects	Sound design	Taxonomy	Data-driven	17
Sigman and Misdariis (2019)	alarm/will/sound: Sound design, modeling, perception and composition cross-currents	Sound design	Taxonomy	Theoretical	56
Nguyen et al. (2006)	First Steps to an Audio Ontology-Based Classifier for Telemedicine	Medicine	Ontology	Theoretical	8
Dimoulas et al. (2011)	Pattern classification and audiovisual content management techniques using hybrid expert systems: A video-assisted bioacoustics application in Abdominal Sounds pattern analysis	Medicine	Taxonomy	Theoretical	13
Boucher et al. (2013)	Identification of common lung sound abnormalities and associated pathologies	Medicine	Taxonomy	Theoretical	12

The number (N) of classes is summed across categorization schemes from the same publication. ID, identification label for multiple categorization schemes, part of the same resource.

### Information extraction and sound descriptor analysis

For every study included in the review, the following information was extracted: first author, title, year of publication, research field, and reasoning (i.e., data-driven or theory-driven). We then computed the total number of classes in each taxonomy/ontology. The subordinate levels of some taxonomies (Bones et al., 2018, p. 3), included repeated classes that referred to different sounds exemplars of the same class presented in the behavioral experiments (e.g., “dog1,” “dog2,” etc.). These exemplar-level repetitions were excluded from the analyses (e.g., only “dog1” was kept).

**Figure 1:**
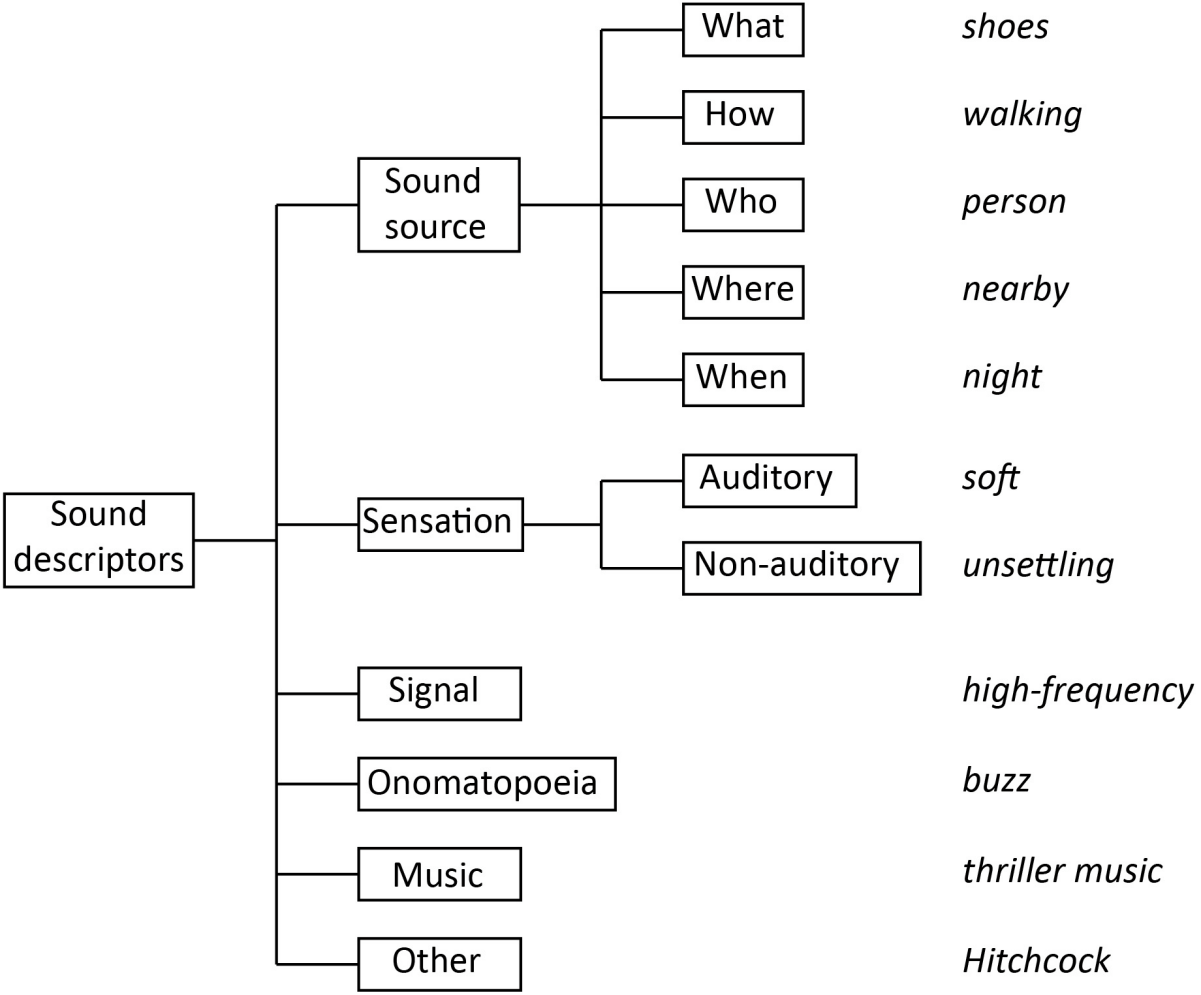
Hierarchy of sound-descriptor categories considered to characterize the semantics of the surveyed taxonomies and ontologies of everyday sounds (examples in italic).

We then carried out an analysis of the semantic domains covered by the class definitions by assigning taxonomy/ontology class descriptors to the following categories (classification carried out by authors RdMA and BLG; see Figure 1):

(a) *Source*—describing the attributes of the sound-generating objects and events, and comprising the following attribute classes:

(a.i) *what*—denoting the vibrating object and substance that generate the sound (e.g., shoes);
(a.ii) *how*—denoting the actions or mechanisms of sound generation (e.g., walking);
(a.iii) *who*—denoting the sound-generating agent (e.g., person);
(a.iv) *where*—denoting the spatial context (e.g., in a train station);
(a.v) *when*—denoting the temporal context (e.g., in the morning; during a holiday).

(b) *Sensation*—describing perceived or recognized attributes of the sound, and comprising the following attribute classes:

(b.i) *auditory*—denoting auditory sensation attributes (e.g., “loud,” “dull”);
(b.ii) *non-auditory*—denoting non-auditory attributes (e.g., “beautiful,” “relaxing,” and also “alarm” and “alert”).

(c) *Signal*—describing sounds at the signal level, and encompassing acoustic descriptors (e.g., “4 Hz modulation energy”), signal-processing terminology (e.g., “Mel-scale frequency cepstral coefficients,” “flanger”), music theory terminology (e.g., “chord,” “harmony”), and synthetic sound-signal classes (e.g., “sfx,” “sound effect,” “effects,” “noise,” “artificial sound,” “textures”).
(d) *Onomatopoeia*, a special class of sound descriptor whose phonation produces vocal sounds that resemble those it denotes (e.g., “buzz”; e.g., Assaneo et al., 2011).
(e) *Music—*comprising the musical-genre descriptors part of the domain-general taxonomies/ontologies selected for this survey.
(f) *Other*—referring to labels that do not describe a sound (e.g., “organization,” “document”).

Each of the above six descriptor categories included a *generic* sub-category that included the category name (e.g., “signal”) and a series of descriptors that could not differentiate among class exemplars (e.g., “natural sound,” “auditory scene,” “soundscape” for the sound source category; “perception,” “experience,” for the category of sensation descriptors).

Each class definition could contain multiple sound descriptions (e.g., multiple class-defining exemplars as “crumpling, crushing” in Gaver, 1993) and each sound description could contain multiple words (e.g., “train station” in Salamon et al., 2014). With the notable exception of several generic descriptors defined by multiple words (e.g., “sound source,” “audio event”), the sound-descriptor classification was in general carried out on a word-by-word basis, at the level of single verbs, nouns, adjectives and adverbs in the class definitions. Each word could potentially be assigned to multiple descriptor categories (e.g., “earthquake” was classified both what/how), with eventual arising ambiguities resolved within the context of each taxonomy/ontology (e.g., “pop” in “pop music” was classified as a music genre, whereas “pop” in the class definition “burst, pop” was classified as how/onomatopoeia).

## Results

### Systematic search

**Figure 2:**
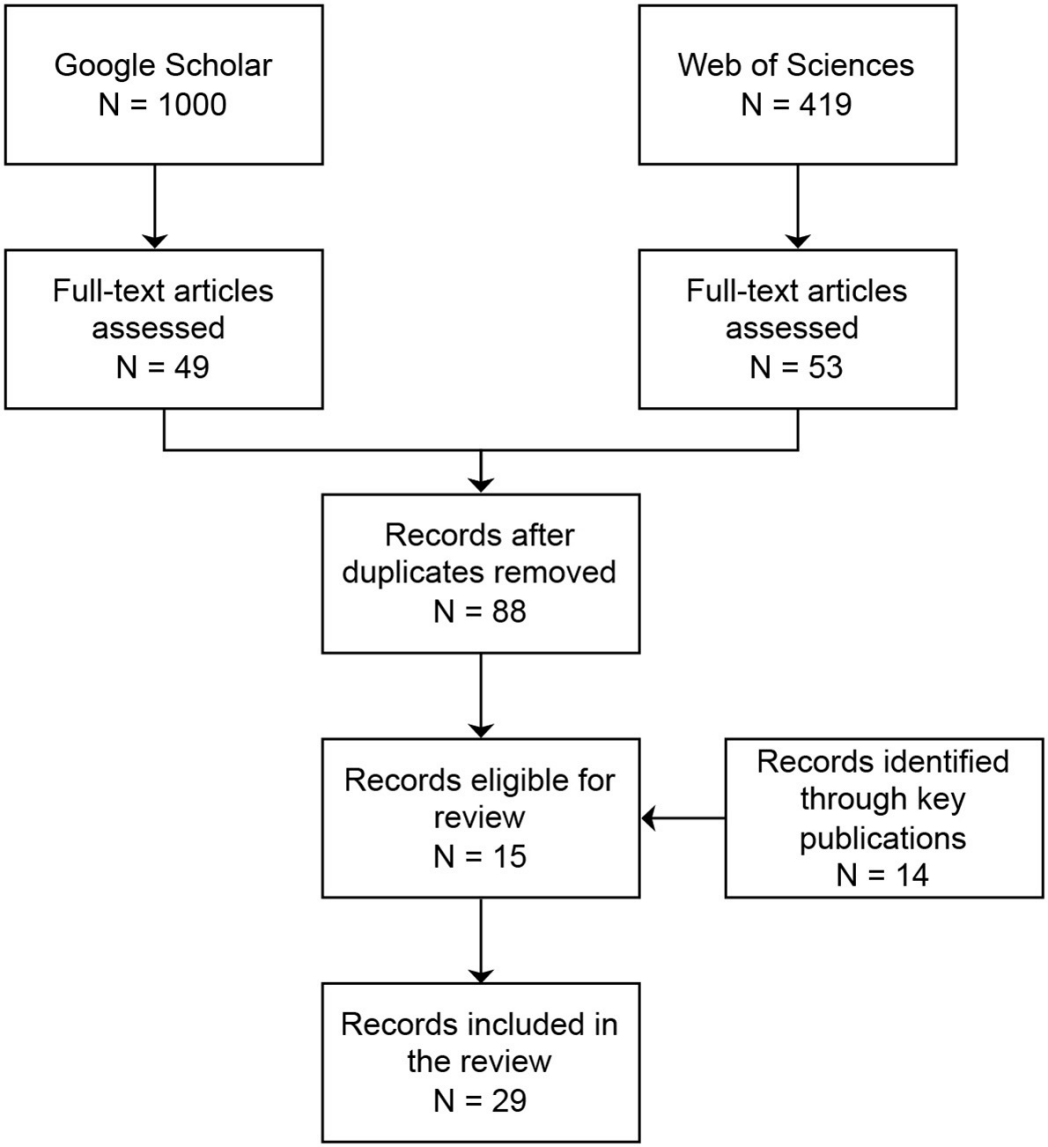
Manuscript selection flow chart.

The systematic search in Google Scholar and Web of Science yielded a total of 1,000 and 419 hits, respectively. After screening the abstracts, 49 records were selected from the results of Google Scholar and 53 records were selected from Web of Science, resulting in 88 unique records after removal of duplicate records. After full-text screening, 15 records were included for review. Additionally, 14 records were identified through references in key publications about sound taxonomies and ontologies, for a total of 29 records through the systematic search (see Figure 2, for a flow chart of the inclusion of records and Table 1 for the list and characteristics of the selected records).

The 29 recovered records spanned across five different research fields: *auditory cognition* (N = 7 records, 24%), *machine hearing* (*N* = 7, 24%), *soundscape* research (*N* = 7, 24%), *sound design* (*N* = 5; 17%), and *medicine* (*N* = 3, 10%). Across the 29 records, 3 publications (10%) reported more than one knowledge organization system, for a total of 32 taxonomies (89%) and 4 ontologies (11%). Three of the selected records described a taxonomy (i.e., included only hierarchical categories), but were labeled as ontologies by their authors (Nakatani and Okuno, 1998; Nguyen et al., 2006; Gemmeke et al., 2017).

### Sound descriptor analysis

The 36 knowledge organization systems included a total of 2,319 classes (average number of classes per system = 64.42; SD = 113.69; median = 30.5), 2,567 sound descriptions (average number of descriptions per class = 1.11; SD = 0.36; median = 1.0), and 3,684 words (average number of words per description = 1.43; SD = 0.77; median = 1.0; N unique words = 1,563).

**Figure 3:**
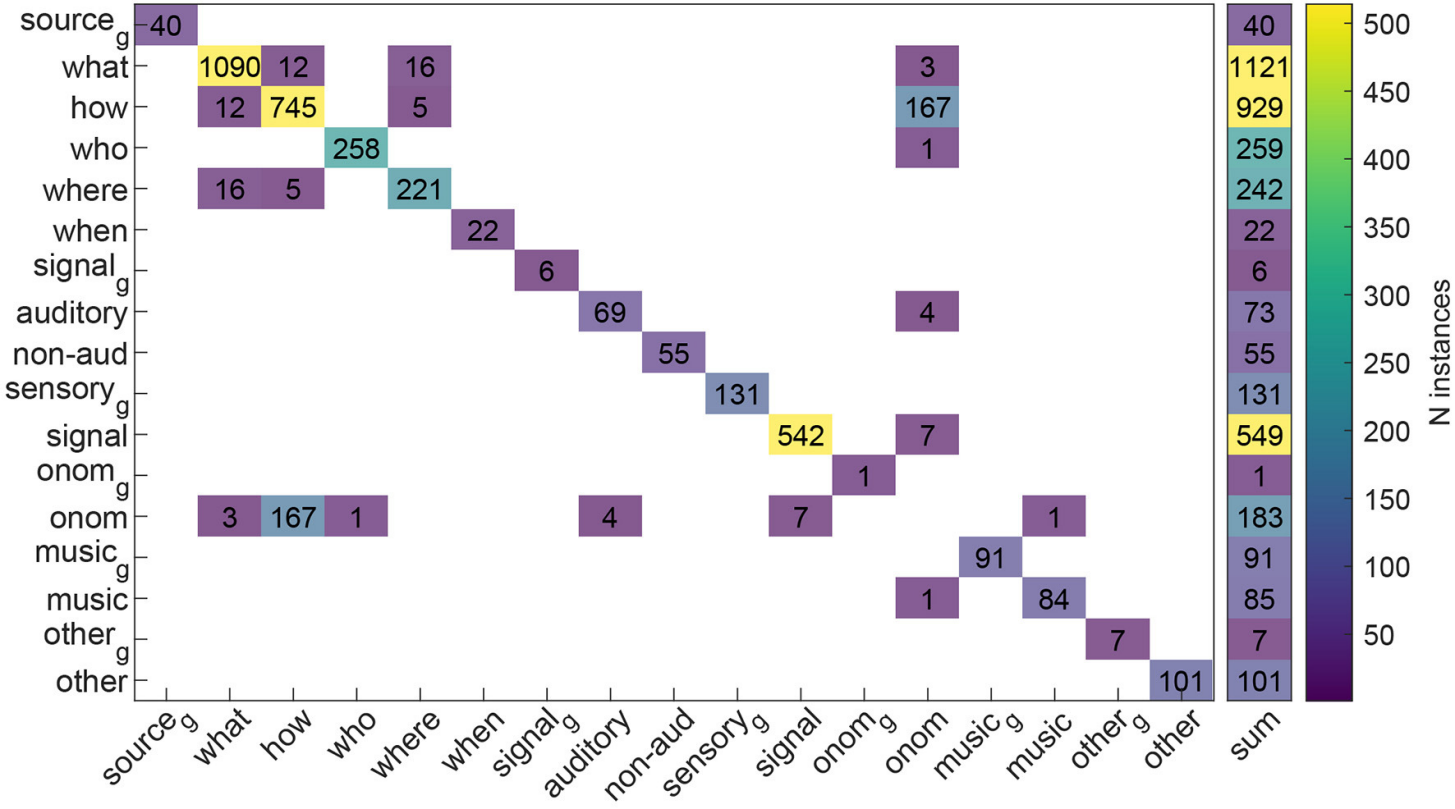
Co-occurrence of sound-descriptor word-by-word classifications across all considered knowledge organization systems. Off-diagonal values indicate words classified, simultaneously in more than one descriptor class, whereas diagonal values indicate word classifications attributed to one single class. The subscript g denotes the generic descriptor classes (e.g., “sound source,” “audio signal”); non-aud, non-auditory; onom, onomatopoeia.

The vast majority of the words (3,478, 94.41%) were assigned to one single sound descriptor class (see Figure 3). The remaining 206 words (5.59%) were instead assigned to more than one descriptor class. Most of these 206 “plurivalent” word classifications were onomatopoeias (183, 88.84%) that denoted in the majority of the cases the properties of a sound-generating action (e.g., “crack,” “woosh”; how/onomatopoeia ambivalence; *N* = 167, 81.07%). The rest of the plurivalent word classifications (23, 16.02%) included words classified as belonging to multiple subclasses of the sound-source descriptors: sport names (“bowling,” “ping-pong”; *N* = 5, 2.47%) that specified the what, how and where sub-attributes of the sound source, what/where ambivalences such as “rail” and “road” (*N* = 11, 5.34%), and “what/how” ambivalences (e.g., “earthquake”; “heartbeat”; *N* = 7, 3.40%).

**Figure 4:**
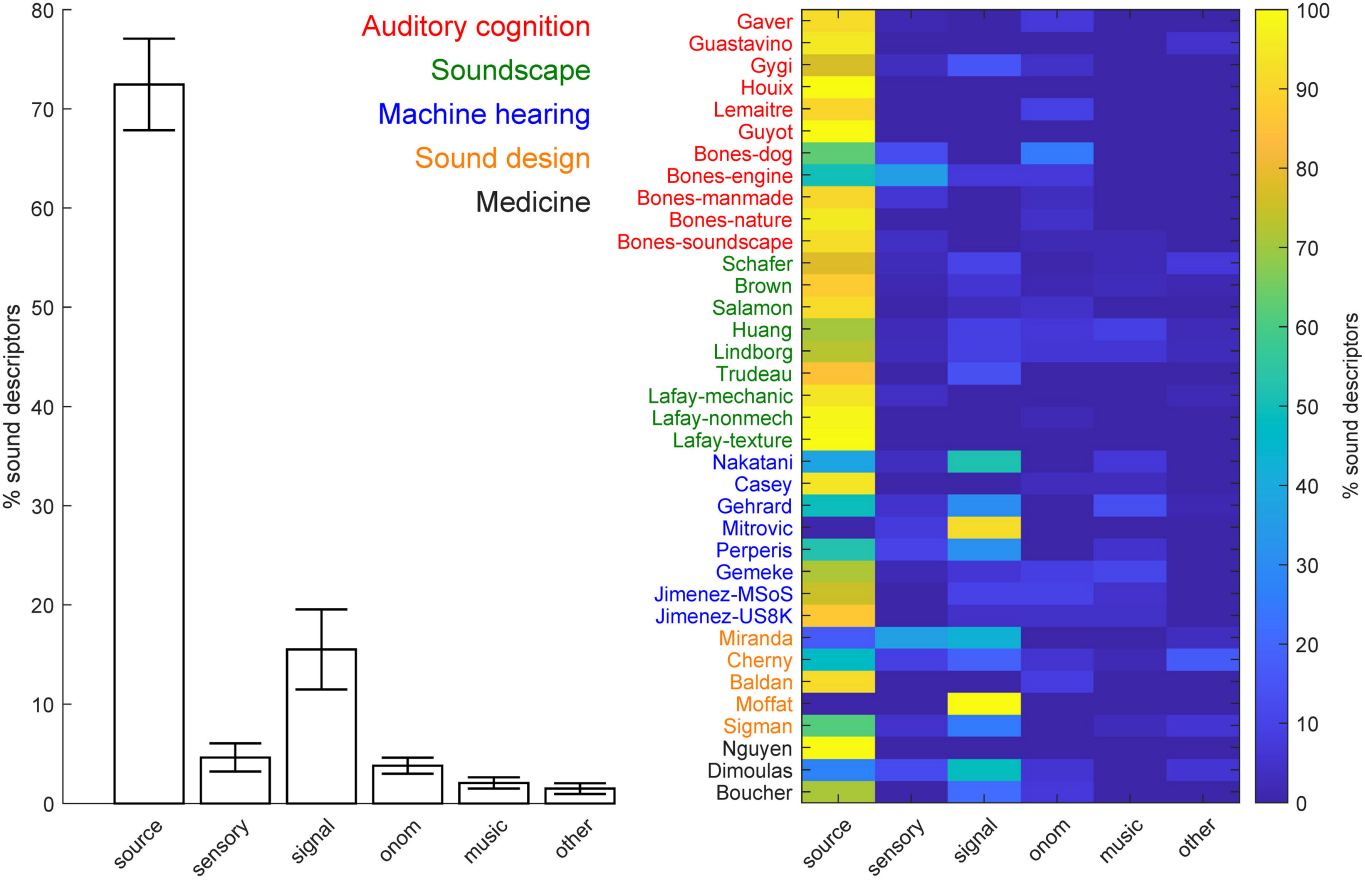
Percentage of sound descriptors in each of the five main categories, including the generic descriptors. Left = average % across knowledge organization systems (error-bar = ± 1 SEM).

**Figure 5:**
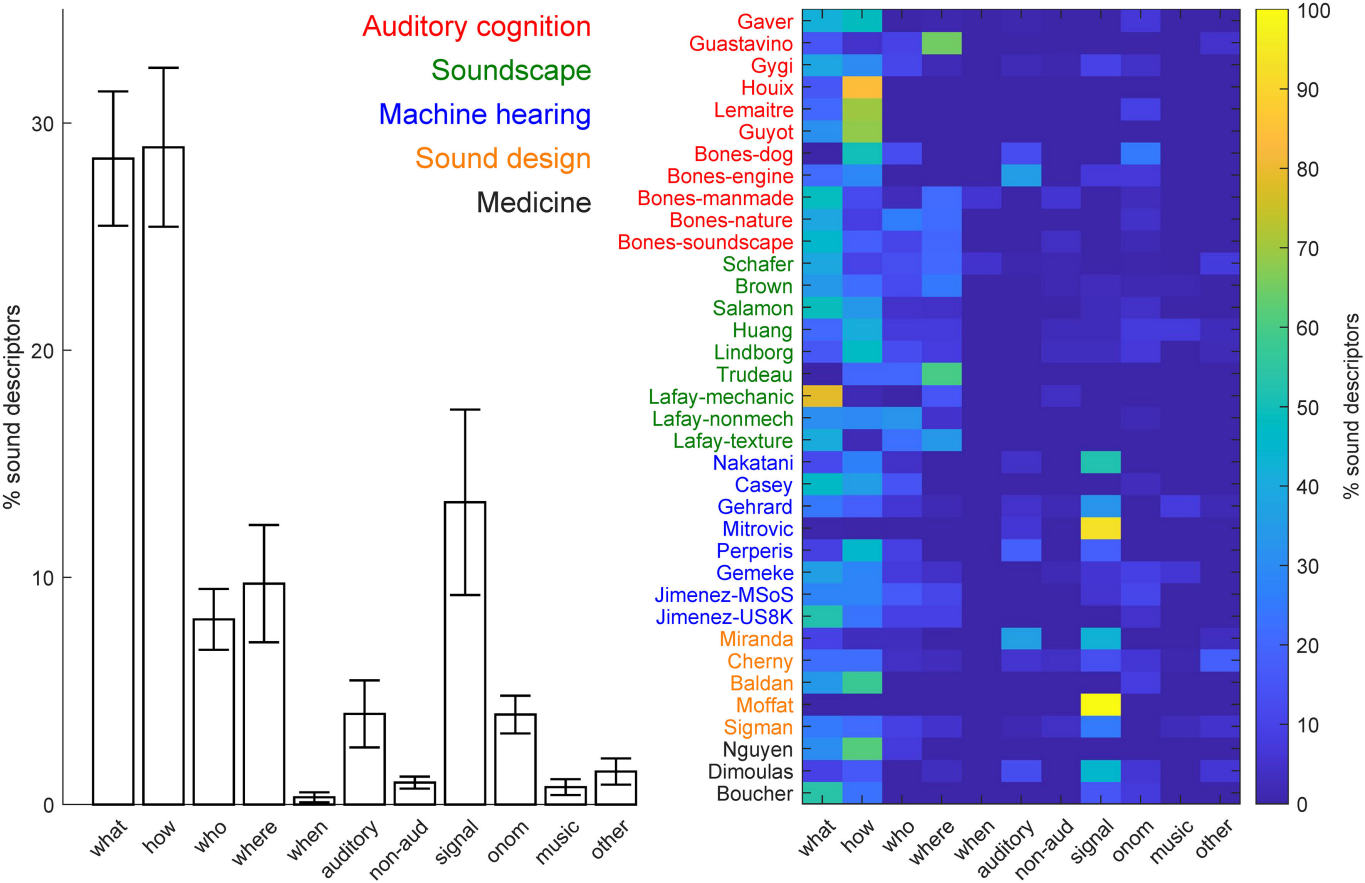
Percentage of sound descriptors in the different categories and subcategories after exclusion of the generic descriptors. Left = average % across knowledge organization systems (error-bar = ± 1 SEM).

We initially focused on the percentage of sound descriptors in each of the five main categories, including the generic descriptors (see Figure 4). Most of the taxonomies/ontologies were hybrid, i.e., included descriptors from multiple categories (N = 31, 86.11%). Five of the knowledge organization systems (13.89%) included, instead, descriptors from one class only, with four including only sound-source descriptors (Nguyen et al., 2006; Houix et al., 2012; Guyot et al., 2017; Lafay et al., 2018), and one (Moffat et al., 2018) including only signal descriptors. The majority of the systems (*N* = 31, 86.11%) comprised, mostly, sound-source descriptors (average % across systems = 72.45%, SEM = 4.61%). The remaining systems (*N* = 5, 13.89%) instead comprised mostly signal descriptors (average % across systems = 15.51%, SEM = 4.03%; see Figure 4 for details). We then considered the percentage of descriptors in the various categories and sub-categories, after excluding the superordinate descriptors (see Figure 5). On average, sound source descriptors comprised mostly *what* and *how* descriptors of the sound-generating objects and actions, respectively (average % across systems = 28.44 and 28.94%, respectively, SEM = 2.96 % and 3.50%), followed by *who* and *where* descriptors of the agent and spatial context for the sound generation, respectively (average % across systems = 8.15 and 9.72%, respectively, SEM = 1.34 and 2.58%). Descriptors of the temporal context for the sound generation (*when*) were the least represented (average % across systems =0.32%, SEM = 0.22%).

**Figure 6:**
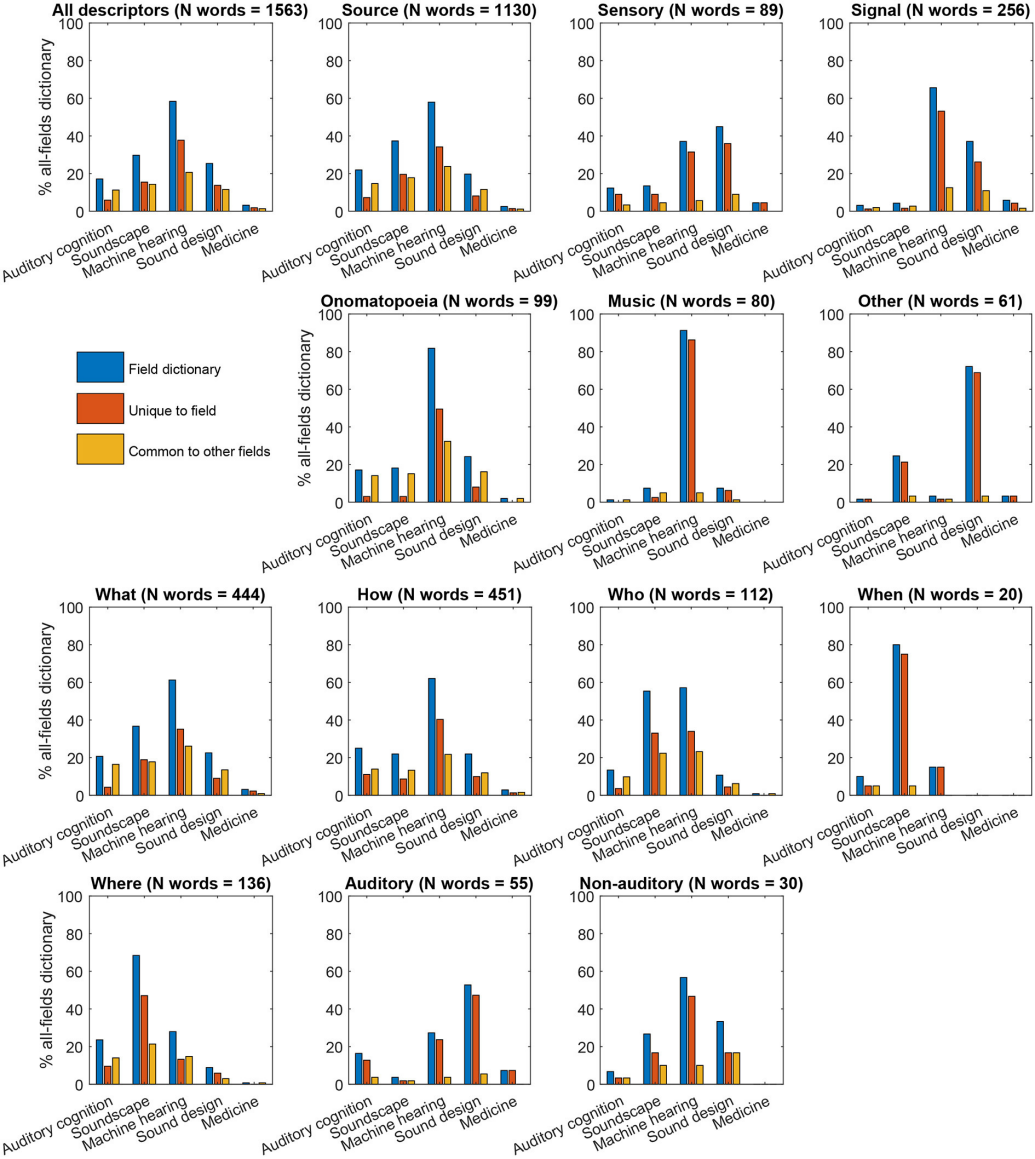
Percentage of sound descriptors in the various semantic categories (Figure 1) included in the knowledge organization systems developed within the reviewed research fields.

We finally carried out a dictionary-overlap analysis to quantify the contribution of each research field to the verbal description of natural sounds in each of the semantic categories of the hierarchy of natural sound descriptors (Figure 6). Machine hearing systems included the largest number of natural sound descriptors for the entire dictionary (N unique words = 1,563), for the majority of the main classes of sound descriptors (*source, signal, onomatopoeia, music*, and *other*, N unique words = 1,130, 256, 99, 80, and 61, respectively), and for the majority of the sound descriptor subclasses (what, how, who, and non-auditory subclasses, N unique words = 444, 451, 112, and 30, respectively). This, in general, expressed the fact that machine hearing systems comprised, overall, the largest dictionary of sound descriptors (N unique words = 913; N unique words for systems in auditory cognition, soundscape research, sound design, and medicine = 268, 465, 396, and 50, respectively). More interestingly, systems developed within the field of sound design contained the largest dictionary of *sensory* descriptions (N unique words = 89) and, more specifically, for the description of the *auditory* attributes of the heard sounds (N unique words = 55), whereas systems developed within the field of soundscape research included the largest dictionaries for the description of the spatial and temporal sound source context (*where* and *when*, N unique words = 136, and 20, respectively).

## Examples of taxonomies and ontologies in sound research

### Auditory cognition

The theoretical framework introduced by Gaver in the early 1990's has been very influential for subsequent auditory cognitive research on real-world sound perception (Gaver, 1993). Gaver considered listening to everyday sounds from the perspective of its ecological goal, i.e., source inference, rather than from a merely perceptual perspective. He produced a taxonomy of everyday sounds based on *audible source attributes*, linked to the physical properties of the sources and to the physical mechanisms of sound generation and not on common auditory perceptual dimensions. The main categorization in Gaver's taxonomy thus entails the material of the sound producing sources: vibrating (solid) objects, aerodynamic sounds and liquid sounds. The subsequent hierarchical tree refers to simple material-specific physical mechanisms (e.g., impact, scraping, for solid material, explosions for gasses, and dripping, splashing for sounds) and to more complex and hybrid events that combine different materials and distinct physical mechanisms (e.g., rain on a surface, drip in a container). The taxonomy is rather limited (it has a total of 56 classes), but it has the merit of highlighting the relevance of physical mechanisms (*how*) in the description of everyday sounds.

Whereas Gaver's work was purely theoretical, records labeled in our survey as behavioral research included mostly taxonomies derived through experimental studies where participants are asked to listen to multiple sounds and to group them according to their perceptual similarity. In these studies, authors aim to understand how humans perceive and categorize sounds at different levels of semantic abstraction. In a recent study, Bones et al. (2018) derived five different taxonomies from their behavioral experiments, conducted through a web application. Participants were asked to listen to different sounds and sort them into five arbitrary groups, which they could name as they wish. For each of the five experiments, authors derived taxonomies by hierarchical cluster analysis of participants' responses. The “top level” of their taxonomies, the “soundscape” taxonomy, represents sounds described in the soundscape literature, including multiple indoor and outdoor environments. The taxonomy has three classes on its superordinate level: “man-made,” “nature” and “people.” The class “people” has the subclasses “voices” and “music.” Nature has the subclasses “animals” and “weather” and man-made has the subclasses “industrial” and “household.” The subordinate level includes the audio samples used in the behavioral task (e.g., “children,” “crowd” as subclasses of “voices” or “water 1” or “thunder 1” as subclasses of “weather”). This taxonomy has in total 50 classes. From this top-level “Soundscape” taxonomy, two categories were selected for further exploration in a new experiment: “man-made” and “nature.” Two middle level taxonomies were derived from each experiment. The “nature” taxonomy has three classes in the superordinate level: “animals,” “water” and “nature” and includes 21 classes (after cleaning repeated classes in the subordinate level). The “man-made” taxonomy has two subordinate classes: “home” and “outside” and includes 28 classes. The article also includes two additional taxonomies, addressing selected sound classes (“dogs” and “engines”) generated through hierarchical clustering of participants' responses. A similar specific level of sound categorization was addressed in Guyot et al. (2017) describing an experiment to understand how humans categorize liquid sounds, through a sorting task performed by 30 participants. The participants had to create classes of liquid sounds according to their physical similarities. By means of agglomerative cluster analysis, the results were subsequently grouped in a taxonomy describing continuous and discrete interactions of solids and liquids.

### Soundscape research

Brown and colleagues defined a taxonomy for soundscape studies reflecting mostly the outdoor, urban sonic environment (Brown et al., 2011). With a total of 47 classes, this taxonomy was built with the goal of generating a common framework for soundscape research and of promoting interoperability between different research fields. The taxonomy originated from a working group conducted in 2009 (“assessment of soundscape quality”), where participants from multiple disciplines were involved, such as acoustics, engineering, planning, architecture, design, park management, psychology, sound quality, sociology and geography. The classes of this taxonomy were based on two criteria: I- It can be applied to any acoustic environment, and II- the way the classes were named aimed to avoid judgements of value or double connotations. The “acoustic environment” class is located at the superordinate level, and is parent of the classes “Indoor” and “outdoor” acoustic environments. The taxonomy is focused on the class “urban acoustic environment” and, at its subordinate level, it includes classes such as “rail traffic, roadway traffic, marine traffic and air traffic,” all under the same parent class “motorized transport.” Another example of subordinate classes are “bells,” “clock chimes,” “fireworks,” “azan” and “alarms,” all subclasses of the parent class “social/communal.”

Lindborg (2016) provides a recent example of soundscape taxonomy aimed at describing sounds of a specific environment, such as restaurant sounds. This taxonomy could assist in developing tests for soundscape research (e.g., regarding pleasantness), or for developing sound simulations of restaurants (Lindborg, 2016). Moreover, restaurant owners could use these findings to tailor the soundscapes in their restaurant and improve customer experience. This taxonomy is data-driven, as it is based on data collected in on-site experiments, using free-form annotations and a questionnaire targeting customer behavior and environment perception at restaurants. Data from 40 different restaurants were collected, with a total of 393 subjects participating in the study. The taxonomy was built following cladistics (from the Greek “klados”, branch). Cladistics considers a taxon as the unit that can be associated with the same unit at a higher level. This posed a challenge for our modeling, as a class cannot be parent and child at the same time in ontologies. We therefore computed an extra class, indicating that it is the same class connected to the repeatable parent (e.g., laughter -> laughter.laughter instead of laughter->laughter). We converted Lindborg's taxonomy in an ontology file (.OWL) based on the pictorial representation reported in the manuscript, and estimated 58 classes.

### Machine learning and artificial hearing

Mitrovic et al. (2010) performed an extensive review of the literature on automated content-based audio retrieval, with the purpose of summarizing and organizing the (lower level) audio features that have been used. Audio features can be defined as a digital form of information representation retrieved from the original audio signal. For instance, low-level features incorporate parameters like fundamental frequency, bandwidth or mel-frequency cepstral coefficients (MFCCs). This taxonomy was tailored to assist researchers in selecting (groups) of features appropriate to the specific audio retrieval task and classifies the audio features based on their extraction process and on the domain to which the audio features belong to. In total, this taxonomy has 87 classes. The description of each of these classes is beyond the scope of the present manuscript, and thus just a short overview is provided here. At the higher level of the hierarchy, the following classes are proposed: *temporal domain, frequency domain, cepstral domain, modulation frequency domain, eigen domain* and *phase space*. Commonly used features are included in the temporal and spectral domain. Subclasses of the temporal domain are amplitude, power and zero crossing. The subclasses of the frequency domain are distinguished in physical and perceptual subclasses. The physical subclasses include features such as adaptive time-frequency decomposition, autoregression coefficients and short-time Fourier transformation. The subclasses of the perceptual class are related predominantly to psychoacoustic features, such as brightness, chroma, harmonicity, loudness, pitch and tonality. In the subordinate level of the latter are the subclasses group delay, spectral flux, spectral peaks, spectral slope and subband energy ratio.

**Figure 7:**
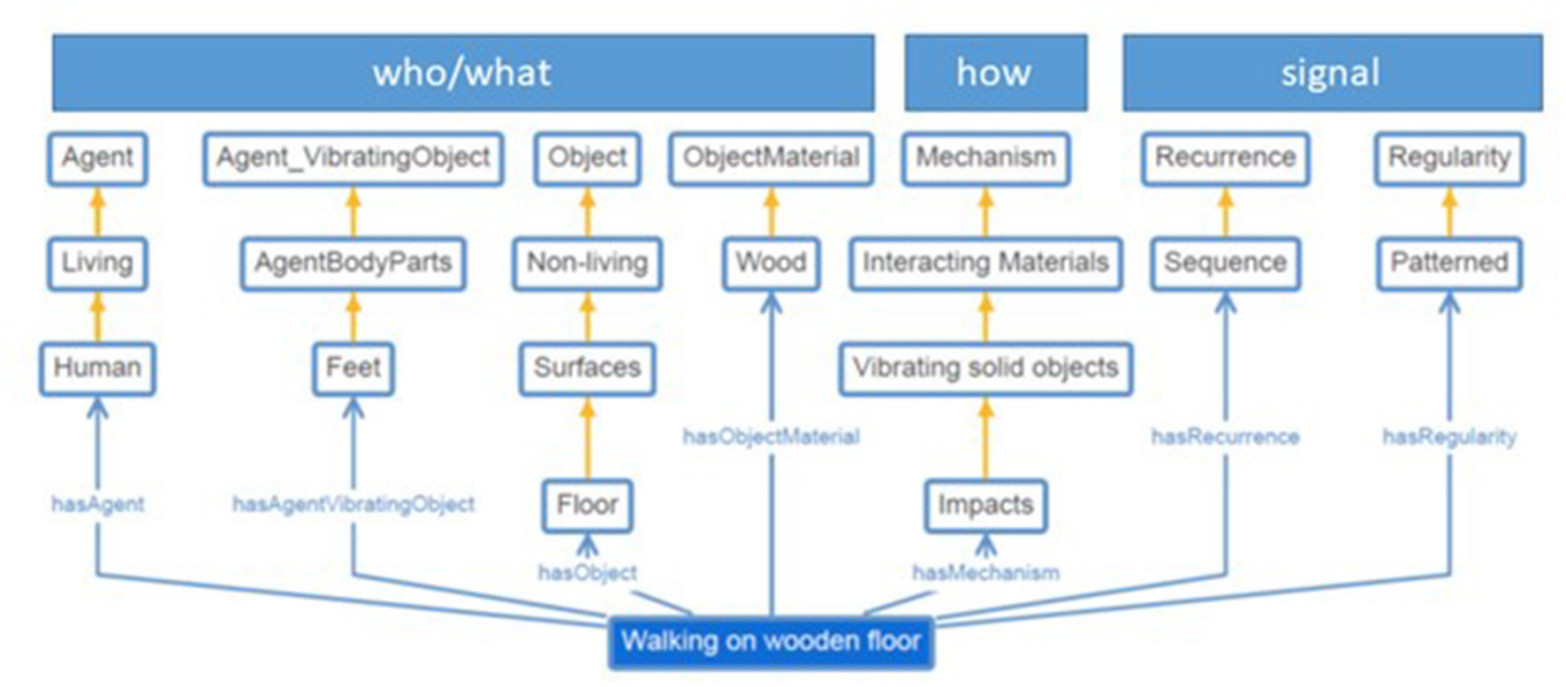
Example of ontological characterization of a “walking on wooden floor” sound event in an ontology that combines descriptions at the sound source and signal levels.

Whereas, the taxonomy by Mitrovic et al. (2010) is mostly concerned with low level acoustic features, the Audioset ontology, published by the Google Research group in 2017 (Gemmeke et al., 2017) organizes real-world sounds, based on their high level (semantic) relation (see Figure 7). AudioSet consists of a hierarchical taxonomy, which is openly accessible, and a dataset of more than 2 million 10 s sound clips drawn from YouTube videos. The project is aimed at assisting the development of sound classification models. The full ontology has 632 classes. A subset of 527 and 523 classes were then used as labels for training the VGGish and Yamnet DNN models (Hershey et al., 2017), respectively. In Audioset, sound classes are subdivided across the following superordinate levels: Human sounds, Animal sounds, Natural sounds, Music, Sound of things, Source-ambiguous sounds, Channel environment and Background. In the subordinate levels, classes like biting, cacophonia or raindrops can be found. Whereas, the large majority of classes has a single parent, a few classes have multiple parents. Classes are linked to URLs from WordNet or Wikipedia. Furthermore, individual instances were added to the model containing the links to the YouTube video. For instance, biting is a subordinate class, and it contains a rdfs:comment with a WordNet URL. It contains the following instances: Biting1 until Biting6. Each of these Biting instances has a URL of a YouTube video with biting sounds.

### Sound design

Despite being a relatively new field, sound design ranked third for number of records (Cherny et al., 2016) built an ontology to optimize searches in very large audio sample databases. Typically, sound designers do this by querying an audio sample management software, which relies on limited, unstructured metadata and generally returns multiple results to be inspected. As that is time-consuming, the authors engineered an ontology that enables structural queries through semantic classes. For instance, queries that can find a specific sound described by the onomatopoeia “whoosh.” To develop the ontology, the authors restructured existing textual metadata of sound samples databases, analyzed various knowledge sources and examined the needs of sound designers through personal interviews. This ontology has 283 classes and deals both with basic metadata (file and library names, etc.) as well as semantic content (*topics*). Among these topics, the authors distinguished classes such as: Sound mood (“arcade,” “orchestral,” “organic”); Sound form (“jingle,” “button,” “slide”); Materials (“human,” “paper,” “glassy”); Source size (“tiny,” “small,” “medium”); interaction type (“negative, “normal,” “positive”). The ontology was also interlinked to a common knowledge ontology (OpenCyc).

### Medicine

Two medical sound taxonomies were identified through the systematic search, one for abdominal sounds (Dimoulas et al., 2011), and another one for lung sounds (Boucher et al., 2013). In both cases, the authors developed the taxonomies to assist diagnosis and to educate medical staff and students in distinguishing physiological from pathological sounds. The Abdominal Sounds Pattern Analysis (ASPA) Schema is a hierarchical taxonomy to classify abdominal sounds, while accounting for interfering noises, such as respiratory related sounds, ambient noises and interfering heart sounds (Dimoulas et al., 2011). In total, this taxonomy has 13 classes. Boucher and colleagues published a taxonomy of breath sounds and lung auscultation based on their literature review. In their taxonomy, “lung sounds” are subdivided in physiological and pathological sounds, which are further categorized in several subclasses. Boucher's taxonomy has in total 12 classes.

## Discussion

### General considerations

In this article, we surveyed published taxonomies/ontologies of everyday sounds. Through a systematic search in two databases, we identified 36 taxonomies/ontologies originating from 29 publications, which could be mapped to five different research fields: *auditory cognition (n* = *11, 30.5%), soundscape research (n* = *5, 13.8%), artificial hearing (n* = *8, 22.2%), sound design (n* = *5, 13.8%)*, and *medicine (n* = *3, 8.3%)*. Approximately half (*n* = *17, 47%*) of the identified taxonomies/ontologies were developed as theoretical frameworks for the characterization of sounds, while the others *(n* = *19, 53%)* were data-driven, i.e., they were derived through the analysis of behavioral data or the analysis of properties of sounds in databases. The extension of the taxonomies/ontologies, as indicated by the number of classes, differed largely and ranged from a few classes to *n* = 633 classes, included in *Audioset* (Google), the largest taxonomy in our search. Furthermore, the level of precision differed greatly between schemes and, in some cases, also within the same scheme for different sound classes.

To evaluate the identified taxonomies/ontologies within a homogeneous framework, we introduced a set of descriptors (*Source, Sensation, Signal, Onomatopoeia, Music, Other*), which we then used to label all extracted classes (*n* = 2,319). The resulting quantitative analysis showed that none of the taxonomies/ontologies covered these different descriptors at the same level of detail. The large majority of taxonomies/ontologies were developed to characterize higher-level semantic relations between sound sources. This suggests that causal listening (Schafer, 1977, or everyday listening as defined by Gaver, 1993) is a default listening mode for natural sounds. More precisely, it shows that the most informative way to describe natural sounds verbally focuses on the properties of the sound source, rather than on sensory or acoustic attributes, or an onomatopoeic dictionary. Examination of the sound descriptors that further specify the source properties indicated that the super-ordinate organization of animate (*who*) and inanimate (*what*) objects was based on commonalities either of the mechanisms of sound generation (*how*) or of the semantic/environmental context (*what*/*where*). The first case is most frequent for taxonomies/ontologies developed in auditory cognitive research (e.g., Gaver, 1993), whereas the latter case is most frequent in soundscape research. *Audioset* follows a taxonomic organization based on the semantic/environmental context for some classes, and on the sound generation mechanisms for other classes. Other taxonomies/ontologies, especially in the research field of artificial hearing and sound design, were concerned with the description of sounds at signal level (e.g., Mitrovic et al., 2010). In these cases, most of the classes described low-level acoustic features, which are directly computable from the sound waveforms, while other classes referred to perceptual auditory dimensions (e.g., pitch, loudness). However, in these taxonomies/ontologies, no formal attempt was made to capture simultaneously all these aspects of the semantic of everyday sounds, or to organize systematically the relation between different semantic aspects such as acoustic-sensory features and higher level semantic properties (e.g., should acoustic-sensory features define the organization of sound exemplars of specific sound sources, or should they constitute an organization scheme orthogonal to that denoting the sound source?). As revealed in an analysis of the overlap of the sound description dictionaries in different fields, a potential route to the development of such a comprehensive ontology of natural sounds will integrate knowledge organization systems across the fields of machine learning (*what* and *how* descriptions), sound design (sensory and auditory descriptions) and soundscape research (*where* and *what* descriptions).

### Open challenges and contribution to future research

**Figure 8:**
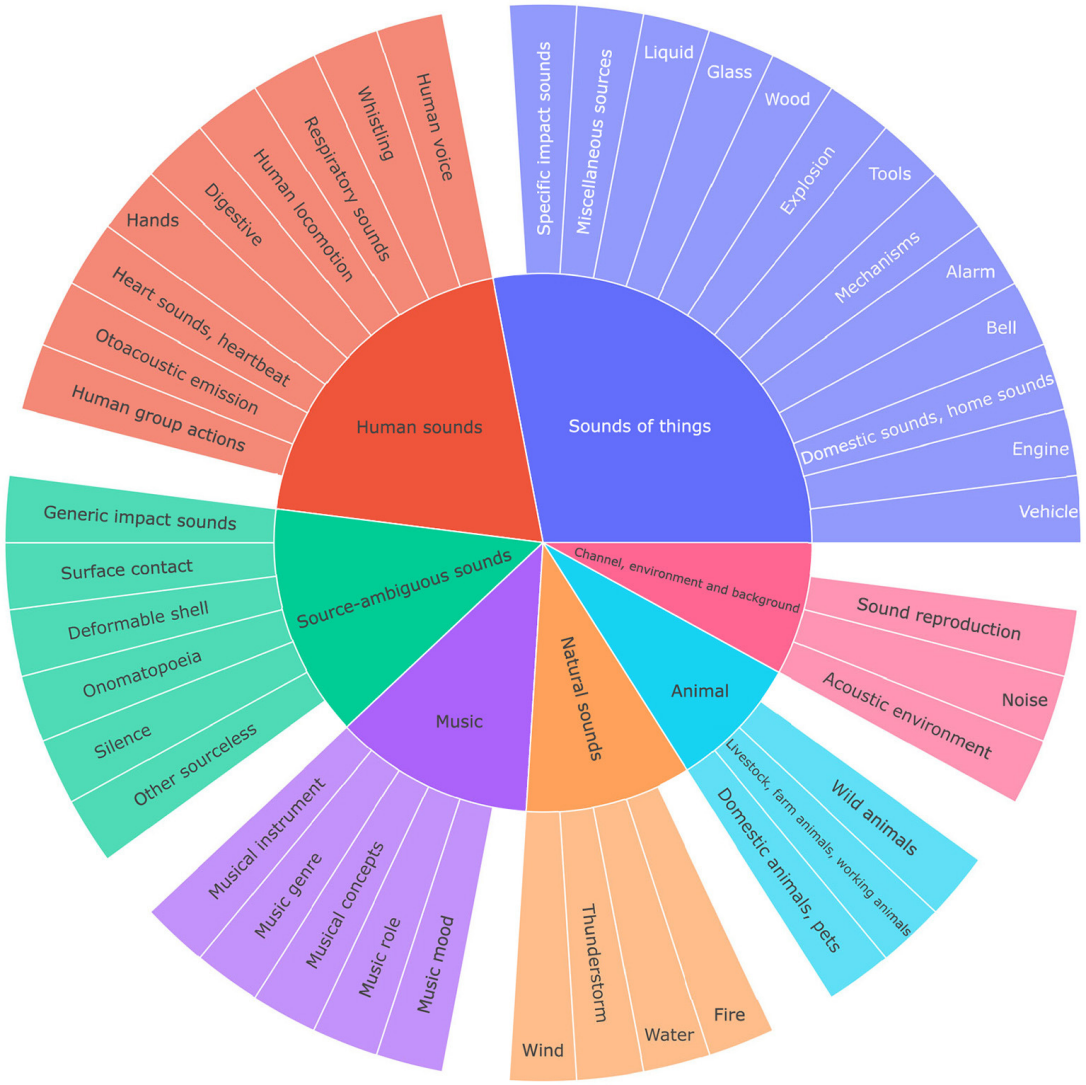
Sunburst diagram representation of the top two levels of the Audioset taxonomy (Gemmeke et al., 2017).

The clear separation between taxonomies/ontologies that cover, at least partially, the high-level semantic properties of sound sources on one side and those covering the sound acoustics on the other side, highlights the lack of a comprehensive ontology covering simultaneously multiple aspects of the sound semantics. A new ontology should describe each sound in terms of a set of properties (as axioms using the Web Ontology Language—OWL), such that an automated OWL-reasoner would be able to check sound description consistency and derive taxonomies automatically. For instance, such an ontology could represent the sound “walking on a wooden floor” as a “patterned sequence (*signal*) of impact sounds generated by the interaction between two solids *(how*: mechanism): the human feet *(who/what*: agent, agent part) and the wooden floor (*who/what*: object).” With these structured descriptions, an OWL-reasoner automatically infers that “walking on a floor” and “tapping fingers on a table,” for example, are both impact sounds (Figure 8).

In this new ontology, the descriptors we introduced could be modeled as sound properties and could be elaborated further. The current set of *who/what* words, as derived from the reviewed studies (see Table 1) is exhaustive and covers a wide range of everyday living and non-living sound-generating objects. However, the set of who/what classes can be easily extended by linking the ontology to existing digital resources, which already include detailed taxonomies of everyday objects (e.g., WordNet, Wikidata). We expect that this approach will result in a collection of objects large enough to cover the majority of applications. Additionally, other object taxonomies developed for specific fields (e.g., musical instruments) or applications (e.g., medical sounds) can be easily incorporated or linked to. The actual required precision of the classes (i.e., if it is acceptable or not that e.g., “seagull” = = “bird”) will then depend on the specific use-case and on concrete aspects, such as the number of labeled sounds in the database for machine hearing applications, or the specific research question for behavioral studies. If needed, classes can be hierarchically propagated to the higher levels to match the desired requirements.

An important challenge for the new ontology will be to express *how* words (typically action verbs and abstract nouns) in terms of elementary sound generation mechanisms, such as those indicated by Gaver (1993) and subsequent psychoacoustic research (e.g., Lemaitre and Heller, 2013). In the example above, “walk” is broken down into a sequence of impacts of solid objects. Performing a similar attribution of properties for all other “how” words will introduce a relevant dimension of sound description, which is not immediately captured neither at signal/acoustic level nor at the level of (linguistic) semantic relations between the sources. To the best of our knowledge, there is no available resource that categorizes verbs and nouns based on the underlying sound-generating mechanisms, thus modeling this property will likely require expert knowledge.

Finally, additional properties in the ontology could be included to describe. contextual information, as that conveyed by *where/when* words. Typically, this information refers either to physical (e.g., “restaurant”) or relative/generic location (e.g., “nearby”) and to temporal contexts (“at night”). An ontology of everyday sounds developed along these lines would capture the multifaceted nature of sound semantics and could be used in several research domains and practical applications, from systems organizing sounds within highly specific domains (such as those encountered in medicine) to the development of multi-task artificial hearing systems capable of captioning sound scenes in terms of objects involved and in terms of pleasantness experienced by the listener. Furthermore, considering the recent trends and direction in AI research, such a resource could be useful to create neuro-symbolic systems for sound analysis (Hitzler and Sarker, 2022). Importantly, using novel ontology embeddings methods (e.g., Chen et al., 2021), numerical representations of sounds and auditory scenes can be derived that encode the semantics described in the ontology graphical and logical structure and in the lexical descriptions. In turn, these numerical sound representations can be used to test and compare hypotheses in model-based analysis of empirical behavioral and neuroimaging data (e.g., Elliott et al., 2013; Giordano et al., 2013; Norman-Haignere et al., 2015; De Angelis et al., 2018).

To favor the integration of the information we gathered in this survey in future developments, we are making available a structured, machine-readable implementation of all taxonomies/ontologies, as well as the entire set of extracted classes (see Data and code availability, below). Only a few of the publications provided a digital version of the taxonomy/ontology and, in most cases, taxonomies/ontologies could only be derived through figures in the article. We reasoned that the availability and easy access to digital versions of the ontologies may help future developments and facilitate their comparison, and enable intercommunication and integration across the research fields. We thus converted each identified taxonomy/ontology in OWL language, which is readable through any text editor and can be edited using ontology editors (e.g., Protegé^®^).

## Data availability statement

Data and owl implementations from this study are available at https://doi.org/10.6084/m9.figshare.20813626. Further inquiries can be directed to the corresponding author.

## Author contributions

BG, EF, and MD: conceptualization, funding acquisition, and supervision. BG and RM: methodology, validation, formal analysis, investigation, data curation, and visualization. BG, RM, YP-C, and EF: software. BG, RM, and YP-C: resources. BG, RM, and EF: writing and original draft. BG, RM, YP-C, EF, and MD: writing and review and editing. EF and MD: project administration. All authors contributed to the article and approved the submitted version.

## Funding

This work was supported by the French National Research Agency (ANR-21-CE37-0027-01 to BG; ANR-16-CONV-0002 – ILCB; ANR11-LABX-0036 – BLRI), the Dutch Research Council (NWO 406.20.GO.030 to EF and MD) and by the Excellence Initiative of Aix-Marseille University (A^*^MIDEX). EF was partially financed by the Dutch Province of Limburg.

## Conflict of interest

The authors declare that the research was conducted in the absence of any commercial or financial relationships that could be construed as a potential conflict of interest.

## Publisher's note

All claims expressed in this article are solely those of the authors and do not necessarily represent those of their affiliated organizations, or those of the publisher, the editors and the reviewers. Any product that may be evaluated in this article, or claim that may be made by its manufacturer, is not guaranteed or endorsed by the publisher.

## References

[B1] Assaneo M. Nichols J. Trevisan M. (2011). The anatomy of onomatopoeia. PLOS ONE. 6:e28317. 10.1371/journal.pone.0028317 22194825 PMC3237459

[B2] Baldan S. Delle Monache S. Rocchesso D. (2017). Sound design toolkit. SoftwareX 6, 255–260. 10.1016/j.softx.2017.06.003

[B3] Bones O. Cox J. T. Davies J. W. (2018). Sound categories: category formation and evidence-based taxonomies. Front. Psychol. 9, 1–17. 10.3389/fpsyg.2018.01277 30104989 PMC6077929

[B4] Boucher N. Prystupa A. Witczak A. Walczak E. Dzida G. Panasiuk L. (2013). Lung auscultation-identification of common lung sound abnormalities and associated pathologies. J. Pre-Clin. Clin. Res. 7, 32–35.

[B5] Brown L. Kang J. Gjestland T. (2011). Towards standardization in soundscape preference assessment. Appl. Acoust. 72, 387–392. 10.1016/j.apacoust.2011.01.001

[B6] Casey M. (2001). General sound classification and similarity in MPEG-7. Organ. Sound 6, 153–164. 10.1017/S1355771801002126

[B7] Chen J. Hu P. Jimenez-Ruiz E. Holter O. M. Antonyrajah D. Horrocks I. (2021). OWL2Vec^*^: embedding of OWL ontologies. Mach. Learn. 110, 1813–1845. 10.1007/s10994-021-05997-6

[B8] Cherny E. Lilius J. Brusila J. Moroumtsev D. Rogozinsky G. (2016). An approach for structuring sound sample libraries using ontology. Int. Conf. Knowl. Eng. Semantic Web. 649, 202–214. 10.1007/978-3-319-45880-9_16

[B9] De Angelis V. De Martino F. Moerel M. Santoro R. Hausfeld L. Formisano E. (2018). Cortical processing of pitch: model-based encoding and decoding of auditory fMRI responses to real-life sounds. Neuroimage 180, 291–300. 10.1016/j.neuroimage.2017.11.020 29146377

[B10] De Coensel B. Botteldooren D. De Muer T. (2003). 1/f noise in rural and urban soundscapes. Acta Acust. United Acust. 89, 287–295.

[B11] Dimoulas C. Papanikolaou G. Petridis V. (2011). Pattern classification and audiovisual content management techniques using hybrid expert systems: a video-assisted bioacoustics application in Abdominal Sounds pattern analysis. Expert Syst. Appl. 38, 13082–13093. 10.1016/j.eswa.2011.04.115

[B12] Elliott T. M. Hamilton L. S. Theunissen F. E. (2013). Acoustic structure of the five perceptual dimensions of timbre in orchestral instrument tones. J. Acoust. Soc. Am. 133, 389–404. 10.1121/1.4770244 23297911 PMC3548835

[B13] Gaver W. W. (1993). What in the world do we hear?: an ecological approach to auditory event perception. Ecol. Psychol. 5, 1–29. 10.1207/s15326969eco0501_1

[B14] Gemmeke J. Ellis D. Freedman D. Jansen A. Wade L. Moore R. . (2017). Audio Set: an ontology and human-labeled dataset for audio events, in IEEE International Conference on Acoustics, Speech and Signal Processing (ICASSP) (New Orleans, LA), 776–780.

[B15] Gerhard D. (2000). Audio signal classification: an overview. Can. Artif. Intellig. 4–6.

[B16] Giordano B. L. Stephen M. Zatorre R. J. Kriegeskorte N. Belin P. (2013). Abstract encoding of auditory objects in cortical activity patterns. Cereb. Cortex 23, 2025–2037. 10.1093/cercor/bhs162 22802575

[B17] Guastavino C. (2007). Categorization of environmental sounds. Can. J. Exp. Psychol. 61, 54–63. 10.1037/cjep2007006 17479742

[B18] Guastavino C. (2018). Everyday sound categorization, in Computational Analysis of Sound Scenes and Events, Eds Virtanen T. Plumbley M. D. Ellis D. (New York, NY: Springer International Publishing), 183–213. 10.1007/978-3-319-63450-0

[B19] Guyot P. Houix O. Misdariis N. Susini P. Pinquier J. Andre-Obrecht R. (2017). Identification of categories of liquid sounds. J. Acoust. Soc. Am. 142, 878–889. 10.1121/1.4996124 28863587

[B20] Gygi B. Kidd G. Watson C. (2007). Similarity and categorization of environmental sounds. Attent. Percept. Psychophys. 69, 839–855. 10.3758/BF03193921 18018965

[B21] Hadley W. Romain F. Henry L. Müller K. (2022). dplyr: A Grammar of Data Manipulation. Available online at: https://dplyr.tidyverse.org (accessed March 1, 2022).

[B22] Hershey S. Chaudhuri S. Ellis D. P. Gemmeke J. F. Moore R. C. (2017). CNN architectures for large-scale audio classification, in 2017 IEEE International Conference on Acoustics, Speech and Signal Processing (ICASSP) (New Orleans), 131–135.

[B23] Hitzler P. Sarker M. K. (Eds.). (2022). Neuro-Symbolic Artificial Intelligence: The State of the Art. Amsterdam: IOS Press, Incorporated.

[B24] Houix O. Lemaitre G. Misdariis N. Susini P. Urdapilleta I. (2012). A lexical analysis of environmental sound categories. J. Exp. Psychol. Appl. 18, 52–80. 10.1037/a0026240 22122114

[B25] Huang L. Kang J. (2015). The sound environment and soundscape preservation in historic city centres—the case study of Lhasa. Environ. Plann. B Plann. Design 42, 652–674. 10.1068/b130073p

[B26] Jiménez A. Elizalde B. Raj B. (2018). Sound event classification using ontology-based neural networks, in 32nd Conference on Neural Information Processing Systems (NeurIPS 2018) (Montreal, QC), 1–9.

[B27] Lafay G. Rossignol M. Misdariis N. Lagrange M. Petiot J.-F. (2018). Investigating the perception of soundscapes through acoustic scene simulation. Behav. Res. Methods 51, 1–24. 10.3758/s13428-018-1138-0 30334149

[B28] Lafay G. Rossignol M. Misdariis N. Lagrange M. Petiot J.-F. (2019). Investigating the perception of soundscapes through acoustic scene simulation. Behav. Res. Methods. 51, 532–555. 30334149 10.3758/s13428-018-1138-0

[B29] Lemaitre G. Heller L. M. (2013). Evidence for a basic level in a taxonomy of everyday action sounds. Exp. Brain Res. 226, 253–264. 10.1007/s00221-013-3430-7 23411674

[B30] Lewis J. W. Brefczynski J. A. Phinney R. E. Jannik J. J. DeYoe E. D. (2005). Distinct cortical pathways for processing tool versus animal sounds. J. Neurosci. 25, 5148–5158. 15917455 10.1523/JNEUROSCI.0419-05.2005 PMC6724809

[B31] Lindborg P. (2016). A taxonomy of sound sources in restaurants. Appl. Acoust. 110, 297–310. 10.1016/j.apacoust.2016.03.032

[B32] Miranda E. Correa J. Wright J. (2000). Categorising complex dynamic sounds. Organ. Sound 5, 95–102. 10.1017/S1355771800002065

[B33] Mitrovic D. Zeppelzauer M. Breiteneder C. (2010). Features for content-based audio retrieval. Adv. Comput. 78, 71–150. 10.1016/S0065-2458(10)78003-7

[B34] Moffat D. Ronan D. Reiss J. (2018). Unsupervised taxonomy of sound effects, in Proceedings of the 20th International Conference on Digital Audio Effects (DAFx-17) (Edinburgh), 428–435.

[B35] Moffat D. Ronan D. Reiss J. D. (2017). Unsupervised taxonomy of sound effects, in Proceedings of the 20th International Conference on Digital Audio Effects (DAFx-2017) (Edinburgh), 428–435.

[B36] Nakatani T. Okuno H. (1998). Sound ontology for computational auditory scence analysissound ontology for computational auditory scene analysis, in Proceedings of the Fifteenth National Conference on Artificial Intelligence and Tenth Innovative Applications of Artificial Intelligence Conference, AAAI 98, IAAI 98 (Madison, WI), 1004–1010.

[B37] Nguyen C. Pham T. Castelli E. (2006). First steps to an audio ontology-based classifier for telemedicine. ADMA 4093, 845–855. 10.1007/11811305_92

[B38] Norman-Haignere S. Kanwisher N. G. McDermott J. H. (2015). Distinct cortical pathways for music and speech revealed by hypothesis-free voxel decomposition. Neuron 88, 1281–1296. 10.1016/j.neuron.2015.11.035 26687225 PMC4740977

[B39] Pachet F. Cazaly D. (2017). A taxonomy of musical genres, in Proceedings of the Content-Based Multimedia Information Access Conference (Paris: RIAO), 1238–1245.

[B40] Perperis T. Giannakopoulos T. Makris A. Kosmopoulos D. Tsekeridou S. Perantonis S. . (2011). Multimodal and ontology-based fusion approaches of audio and visual processing for violence detection in movies. Expert Syst. Appl. 38, 14102–14116. 10.1016/j.eswa.2011.04.219

[B41] Prystupa A. (2013). Lung auscultation – identification of common lung sound abnormalities and associated pathologies. J. Preclin. Clin. Res. 7, 32–35. 10.26444/jpccr/71431

[B42] Salamon J. Jacoby C. Bello J. (2014). A dataset and taxonomy for urban sound research, in Proceedings - 22*nd ACM International Conference on Multimedia* (Orlando), 1041–1044.

[B43] Schafer R. M. (1977). The Tuning of the World. New York, NY: Knopf Ed., 301.

[B44] Sigman A. Misdariis N. (2019). alarm/will/sound: sound design, modelling, perception and composition cross-currents. Organ. Sound. 24, 54–70. 10.1017/S1355771819000062

[B45] Southworth M. (1969). The sonic environment of cities. Environ. Behav. 1, 49–70. 10.1177/001391656900100104

[B46] Trudeau C. Guastavino C. (2018). Classifying soundscapes using a multifaceted taxonomy, in 11th European Congress and Exposition on Noise Control Engineering (Hersonissos), 2487–2492.

[B47] von Hornbostel E. M. Sachs C. (1961). Classification of musical instruments. Galpin Soc. J. 14, 3–29.

